# Development and comprehensive clinical validation of a deep neural network for radiation dose modelling to enhance magnetic resonance imaging guided radiotherapy^☆^

**DOI:** 10.1016/j.phro.2025.100723

**Published:** 2025-02-07

**Authors:** Moritz Schneider, Simon Gutwein, David Mönnich, Cihan Gani, Paul Fischer, Christian F. Baumgartner, Daniela Thorwarth

**Affiliations:** a Section for Biomedical Physics, Department of Radiation Oncology, University Hospital Tübingen, Tübingen, Germany; b Department of Radiation Oncology, University Hospital Tübingen, Tübingen, Germany; c Cluster of Excellence “Machine Learning: New Perspectives for Science”, University of Tübingen, Germany; d Faculty of Health Sciences and Medicine, University of Lucerne, Switzerland; e German Cancer Consortium (DKTK), partner site Tübingen, a partnership between DKFZ and University Hospital Tübingen, Germany

**Keywords:** Dose calculation, MRI-guided radiotherapy, Artificial intelligence, Online adaptive radiotherapy

## Abstract

**Background and purpose:**

Online adaptive magnetic resonance imaging (MRI)-guided radiotherapy requires fast dose calculation algorithms to reduce intra-fraction motion uncertainties and improve workflow efficiency. While Monte-Carlo simulations are precise but computationally intensive, neural networks promise fast and accurate dose modelling in strong magnetic fields. This study aimed to train and evaluate a deep neural network for dose modelling in MRI-guided radiotherapy using a comprehensive clinical dataset.

**Materials and methods:**

A dataset of 6595 clinical irradiation segments from 125 1.5 T MRI-Linac radiotherapy plans for various tumors sites was used. A 3D U-Net was trained with 3961 segments using 3D imaging data and field parameters as input, Root Mean Squared Error and a custom loss function, with full Monte-Carlo simulations as ground truth. For 2656 segments from 50 patients, gamma pass rates (γ-PR) for 3 mm/3%, 2 mm/2%, and 1 mm/1% criteria were calculated to assess dose modelling accuracy. Performance was also tested in a standardized water phantom to evaluate basic radiation physics properties.

**Results:**

The neural network accurately modeled dose distributions in both patient and water phantom settings. Median (range) γ-PR of 97.7% (87.5–100.0%), 89.1% (69.7–99.4%), and 60.8% (38.5–82.1%) were observed for treatment plans, and 97.1% (55.5–100.0%), 88.8% (38.8–99.7%), and 61.7% (17.9–94.4%) for individual segments, across the three criteria.

**Conclusion:**

High median γ-PR and accurate modelling in both water phantom and clinical data demonstrate the high potential of neural networks for dose modelling. However, instances of lower γ-PR highlight the need for comprehensive test data, improved robustness and future built-in uncertainty estimation.

## 1 Introduction

Accurate dose calculation is a crucial step in radiotherapy planning, as the optimization of the treatment plan as well as the estimation of expected tumor control and radiation-induced toxicity depend on it. Monte-Carlo (MC) based dose algorithms are considered the current gold standard for radiation dose simulations in radiotherapy [1], [2]. As MC dose algorithms simulate the particle-wise interaction with matter based on tabulated cross-section data, the accuracy of the simulation is strongly dependent on the number of incident particles [3]. A high number of primary particles leads to increased accuracy in dose simulations, at the cost of computational resources and time [4]. This tradeoff between accuracy and speed still stands, despite technological advances such as the use of graphical processing units (GPU) [5] and dedicated acceleration methods [6]. The computation time for full MC dose simulations using general purpose frameworks is in the order of hours [7], [8], [9], compared to minutes using GPU based implementations [5], [10].

Recent innovations in radiotherapy allow for online adaptation of radiation treatment plans using magnetic resonance imaging (MRI) or computed tomography (CT) guidance [11], [12], [13]. Therefore, the time required for plan adaptation, including dose calculation, should be minimized to reduce positional uncertainties, enhancing the workflow's precision, efficiency, and convenience for the patient [14], [15].

As analytical calculation methods are not able to reflect the complexity of dose calculation in the presence of a magnetic field [16], and due to their speed characteristics, the use of neural networks (NN) for fast radiation dose modelling seems to be very elegant.

Approaches using NNs promise to model dose distributions in several seconds or less with an accuracy comparable to time and resource intensive MC simulations [4], [17], [18], [19], [20], [21]. To date, most of the approaches were developed regarding standard linear accelerators without the effects of a strong external magnetic field on the dose deposition [4], [17], [19], [20], [22], [23]. Kontaxis et al. [19] presented a 3D U-Net allowing for modelling the dose distribution arising from individual irradiation segments. It was trained solely on prostate cancer treatment plans with fixed beams arrangements. The works from Tsekas et al. [24] added an external magnetic field, as well abdominal tumor entities and a VMAT approach was tested on the same dataset as well as few additional treatment plans [25]. Nevertheless, assessment of the capabilities of such networks remained on small [18], [21], [22], homogenous [4], [17], [19], [24], or non-clinical [20] datasets. Since the mechanism of NNs is not per se based on radiation transport principles, careful investigation and characterization of such networks is essential.

This study aimed to develop a robust yet generalized deep dose modeling network by training a 3D U-Net with a customized loss function on a large, heterogeneous clinical 1.5T MRI-Linac dataset, evaluating its accuracy against full MC dose simulations and water phantom measurements.

## 2 Materials and methods

### 2.1 Patient data

We collected a dataset consisting of step-and-shoot IMRT plan data and computed tomography (CT) images of 125 patients treated between 01/2019 and 03/2023 at a 1.5T MRI-Linac (Unity, Elekta AB, Sweden). The study was approved by the institutional review board (659/2017BO1, NCT04172753), all patients gave written informed consent. The dataset included 50 patients with prostate and 25 patients with liver tumors, 25 patients suffering from head-and-neck tumors (HNC), 15 patients with tumorous lymph nodes and 10 patients treated with a partial breast irradiation. The 6649 individual radiotherapy segments from the dataset were split into a training dataset containing 3956 irradiation segments from 75 patients (40 prostate, 15 liver, 15 HNC, 5 partial breast) and a test dataset, completely unseen while training, of 2668 irradiation segment from 50 patients (10 prostate, 10 liver, 10 HNC, 5 partial breast and 15 lymph nodes). By design, tumorous lymph nodes are only present in the test data and are therefore used as out of domain test data. 25 hand-picked irradiation segments served as a held-out validation set.

Full MC-simulations of every beam segment were created as gold standard using EGSnrc [7]. We simulated n = 5E07 primary particles for each segment, ensuring a mean uncertainty per segment of 0.7% [26]. We used an in-house developed beam model for the 1.5T MRI-Linac [27]. The dose grid dimensions were set to 3 × 3 × 3 mm^3^. CT-values were converted to mass densities using the CT-specific calibration curve.

### 2.2 Model architecture

We utilized a 3D U-Net architecture [28], [29] including skip connections. An initial block expands the input from 5 to 64 channels. The encoder then consists of down sampling layers with 3 × 3 × 3 convolutions, batch normalization and ReLU activation, which increase the channel sizes to 64 and 128. In the bottleneck, the channels expand to 256. The decoder uses transposed convolutions to up sample, with channel sizes of 128 and 64, incorporating skip connection at each level. A final 1 × 1 × 1 convolution generates the single channel output.

As an input, patches of 32 × 32 × 32 voxels were sampled from the CT data, with an original CT voxel dimension of 1.17 × 1.17 × 3 mm^3^. Additionally four input masks were created, similar to the methodology described in [19], representing important features for dose modelling. Those masks consist of the MLC-shape propagated through the CT data using raytracing to define the primary irradiation field, as well as the calculated radiological depths, source distances, and distances from the central axis of the respective beam for every voxel in the CT dataset. The ground truth dose grid (3 × 3 × 3 mm^3^) was linearly interpolated to match the voxel dimensions (1.17 × 1.17 × 3 mm^3^) of the CT data.

### 2.3 Model training

A batch size of 128 was used along with a root mean squared error (RMSE) as loss function. A hyperparameter grid search led to the following parameters of the ADAM optimizer: learning rate: 1E−4, beta1: 0.9, beta2: 0.99, epsilon: 1E−8. The validation loss was calculated on 25 hand-picked segments, representing heterogenous segments from all entities present in the training set. After convergence of this training step, a subsequent training using a customized loss function was conducted.

(1) $$L=\sqrt{\frac{1}{n}\sum_{i=0}^{n}\left({\left(y_{i}-\hat{y_{i}}\right)}^{2}+0.1\left({\left(y_{i}-\hat{y_{i}}\right)}^{2}\times G\left(y_{i}\right)\right)\right)}$$

This loss function (Eq. (1)) combines the squared error between ground truth y and prediction $\hat{y}$ at each voxel i with an additional term. This term scales the squared error by the gradient of the ground truth $G\left(y_{i}\right)$, emphasizing regions with higher dose gradients. The gradient calculation is based on a three dimensional Sobel kernel [30]. The kernel can be found in the Supplementary materials, along with an evaluation of the performance of the model without subsequent training. The network was trained until convergence for both loss functions, using four Nvidia RTX2080-Ti GPUs.

### 2.4 Evaluation

For observing the dose distribution for a whole CT dataset, we averaged predictions for overlapping patches with stride 16. To evaluate the performance of the model, a mean dose error was calculated based on all treatment plans of the test dataset. Voxels exceeding 10% of the maximum dose in the treatment plan were included, as otherwise background voxels with low dose would dominate. Further, we conducted a global gamma analysis (3 mm/3%, 2 mm/2% and 1 mm/1%) on voxels above 10% of the maximum dose to assess model and MC simulation agreement [31]. This gamma analysis was performed regarding individual segments of the test set, as well as for treatment plans. To achieve this, the weighted sum of all segment dose maps belonging to one treatment plan was used.

The out of domain lymph node test dataset was tested to evaluate the generalizability of the model. A Mann-Whitney U-Test was utilized to check for significant differences (p < 0.05) between the gamma analysis of the in domain and out of domain test data.

While being trained on irradiation segments of clinical treatment plans, we additionally aimed to evaluate the modelling accuracy in a standardized setting. Therefore, a water equivalent volume was simulated, and the network prediction was compared to relative water phantom measurements (BeamscanMR, PTW Freiburg, Germany) in a 1.5T MRI-Linac (Unity, Elekta AB, Sweden), performed with a microdiamond detector (PTW Freiburg, Germany). The measurements were carried out at the beam isocenter, ensuring a water depth of 10 cm, a gantry angle of 0° and a source-to-surface distance of 133.5 cm. We used a field size of 10 × 10 cm^2^ and analyzed in-line and cross-line profiles, as well as a depth dose curve. The relative measurement was scaled to the mean of the 9 central voxels in 10 cm water depth of the MC-simulated dose. For quantification, a 2D-gamma analysis (2 mm/2%, 10% dose cutoff) between measurement and prediction was conducted.

## 3 Results

**Fig. 1:**
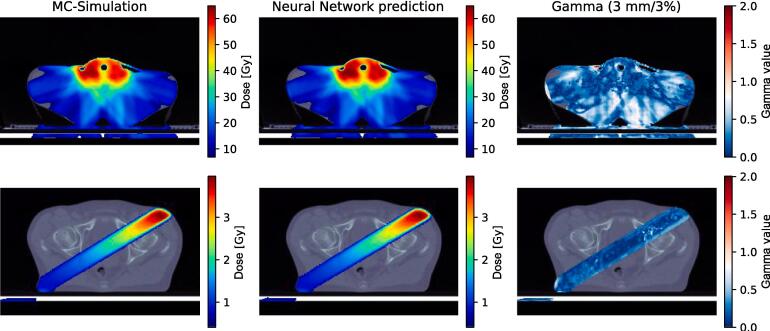
Isocenter slice of the target dose distribution (left), the modelled dose distribution (middle) and the gamma map (3 mm/3%, right) for a treatment plan from the head and neck test dataset (top) and a segment from the prostate test dataset (bottom).

With our model, we were able to generate dose distributions, which agreed with the ground truth data for irradiation segments and total radiotherapy plans for all entities in our test dataset. Exemplary slices of dose distributions of a HNC treatment plan as well as one segment of a prostate plan are shown in Fig. 1. The inference of the network took five seconds per segment on average.

### 3.1 Treatment plans

**Fig. 2:**
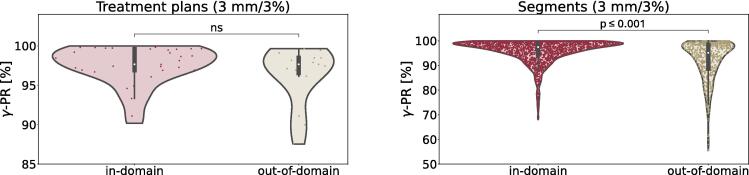
Comparison of in and out of domain treatment plans (left) and individual segments (right) regarding their γ-PR (3 mm/3%). A Mann-Whitney U-Test was used for statistical analysis (significance level: p < 0.05).

For the 50 test set treatment plans, the mean error for voxels above 10% of the MC maximum dose was 0.017 Gy. The median (range) gamma pass rate (γ-PR) using a 3 mm/3% gamma criterion was 97.7% (87.5–100.0%). Separate evaluation of entity-specific subsets in the test dataset demonstrated the highest agreement for prostate cancer treatment plans, reaching a median γ-PR of 99.7% (93.4–100.0%). Median γ-PRs for the subsets which were also represented in the training set, liver, breast and HNC, were 98.4% (96.9–99.8%), 96.7% (94.9–97.4%) and 96.9% (90.2–98.7%), respectively. Inference of lymph node plans, an out-of-domain subset, resulted in a median γ-PR of 97.7% (87.5–99.7%). Using a Mann-Whitney *U* test, the distribution of γ-PRs of treatment plans was not found to be significantly different between the lymph node subset and the other four entities (p = 0.3, Fig. 2). Of the 50 treatment plans evaluated, eight showed a γ-PR below 95%.

**Fig. 3:**
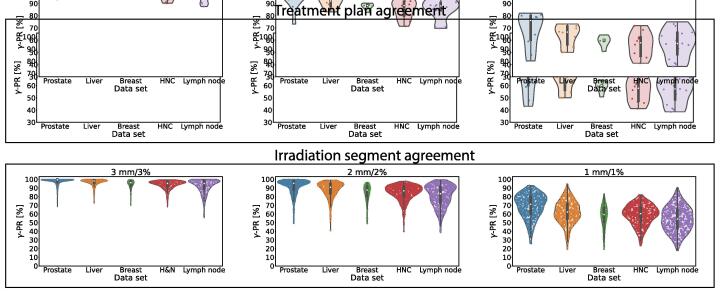
γ-PR (3 mm/3%, 2 mm/2%, 1 mm/1%) for treatments plans (top row) and individual segments (bottom row) from the test set.

Considering the stricter gamma criteria of 2 mm/2% and 1 mm/1%, the agreement decreased to median γ-PRs of 89.1% (69.7–99.4%) and 60.8% (38.5–82.1%) respectively. The different subset's γ-PRs are depicted in Fig. 3 and detailed in Table 1. Note, that the γ-PRs of the different test subsets did not decrease uniformly. The median γ-PR of treatment plans of the prostate test dataset was found to be 97.1% (73.2–99.4%) for a gamma criterion of 2 mm/2%, showing a decrease of 2.6% compared to the 3 mm/3% criterion. The median γ-PR of the lymph node test dataset, in contrast, decreased by 9.7% to 88.0% (69.7–96.8%) considering a 2 mm/2% criterion.

**Table 1:** γ-PRs of treatment plans from the test datasets, evaluated with different criteria, values exceeding 95% in bold.

***Dataset***	*Criterion*	*Median [%]*	*Mean [%]*	*Min [%]*	*Max [%]*	*STD [%]*
***Prostate***	*3 mm/3%*	**99.7**	**99.0**	93.4	**100.0**	1.9
	2 mm/2%	**97.1**	93.8	72.2	**99.4**	7.6
	*1 mm/1%*	76.2	68.9	43.1	82.1	13.7

***Liver***	*3 mm/3%*	**98.4**	**98.4**	**96.9**	**99.8**	1.0
	2 mm/2%	92.1	91.1	83.2	**96.8**	4.8
	*1 mm/1%*	66.6	63.8	50.0	73.3	8.7

***Breast***	*3 mm/3%*	**96.7**	**96.5**	94.9	**97.4**	4.8
	2 mm/2%	88.4	87.6	82.8	90.3	2.5
	*1 mm/1%*	60.1	59.0	50.9	64.3	4.4

***HNC***	*3 mm/3%*	**96.9**	**95.6**	90.2	**98.7**	2.9
	2 mm/2%	88.4	85.2	71.4	94.8	7.5
	*1 mm/1%*	57.8	56.3	41.1	71.9	9.7

***Lymph node***	*3 mm/3%*	**97.7**	**96.3**	87.5	**99.7**	3.6
	2 mm/2%	88.0	85.8	69.7	**96.8**	8.5
	*1 mm/1%*	57.3	57.5	38.5	75.1	11.7

### 3.2 Segments

Evaluating all segments in the test dataset individually with a 3 mm/3% gamma criterion, the median (range) γ-PR was 97.1% (55.5–100.0%). The prostate test set segments showed the highest median (range) γ-PR of 99.4% (68.4–100.0), followed by the liver, 98.0% (72.4–99.9%), partial breast, 96.1% (69.6–99.4%), HNC, 95.3% (68.1–99.5%), and the lymph node test data, 95.1% (55.5–99.9%). In contrast to the evaluation of treatment plans, the Mann-Whitney-U-Test did show a significant difference between the performance of the model on in-domain test data, compared to the out-of-domain test data (p < 0.001, Fig. 2). Despite a high median acceptance rate of >95% for all test data subsets, there were a few segments with low γ-PRs. The minimal γ-PR was, as shown in Table 2, below 75% in all test datasets.

**Table 2:** γ-PRs of individual segments from the test datasets, evaluated with different criteria, values exceeding 95% in bold.

***Dataset***	*Criterion*	*Median [%]*	*Mean [%]*	*Min [%]*	*Max [%]*	*STD [%]*
***Prostate***	*3 mm/3%*	**99.4**	**97.9**	68.4	**100**	3.6
	2 mm/2%	**95.4**	91.9	49.4	**99.7**	8.3
	*1 mm/1%*	71.2	69.1	25.9	92.9	13.8

***Liver***	*3 mm/3%*	**98.0**	**96.6**	72.4	**99.9**	4.1
	2 mm/2%	90.8	88.3	40.9	**98.9**	8.8
	*1 mm/1%*	62.2	62.1	19.3	94.4	13.0

***Breast***	*3 mm/3%*	**96.1**	94.4	69.6	**99.4**	5.4
	2 mm/2%	87.6	84.5	49.1	**96.1**	9.7
	*1 mm/1%*	59.3	57.6	19.4	82.9	12.2

***HNC***	*3 mm/3%*	**95.3**	93.9	68	**99.5**	4.9
	2 mm/2%	86.7	84.8	39.9	**97.7**	8.6
	*1 mm/1%*	60.3	59.0	23.5	82.0	11.6

***Lymph node***	*3 mm/3%*	**95.1**	92.4	55.5	**99.9**	7.8
	2 mm/2%	83.3	81.2	38.8	**99.5**	12.4
	*1 mm/1%*	54.1	54	17.9	90.2	14.4

Utilizing the stricter gamma criteria of 2 mm/2% and 1 mm/1%, the γ-PRs decreased. This decrease is more prominent, compared to the evaluation of treatment plans. The median γ-PR of the test dataset, evaluated with a 2 mm/2% and 1 mm/1% gamma criterion respectively, was 88.8% (38.8–99.7%) and 61.7% (17.9–94.4%), respectively. The distribution of the γ-PRs is shown in Fig. 3.

### 3.3 Water phantom

**Fig. 4:**
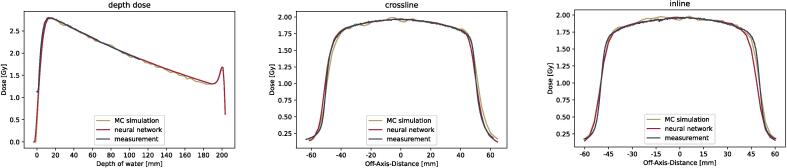
Absolute dose profiles of Monte-Carlo simulation, measurements and neural network prediction for 10 × 10 cm^2^ irradiation field in a water phantom.

Modelling the dose distribution in a water phantom, the form of the depth-dose curve with dose buildup and dose maximum, the dose decrease with increasing water depth and the electron return effect at the back end of the phantom were modelled accurately (γ-PR (2 mm/2%) = 100%). Similarly, the general beam shape was modelled correctly in the cross-line direction (perpendicular to the magnetic field) achieving a 2D γ-PR (2 mm/2%) of 100%. In the in-line direction, slight discrepancies were observed in the penumbra region (γ-PR (2 mm/2%): 90.5%), but still being smaller than one slice thickness of the original dataset (Fig. 4). Statistical noise present in the MC simulations, was not visible in the modelled dose distribution.

## 4 Discussion

In this work we were able to model physically meaningful dose distributions of irradiation segments and clinical treatment plans for a comprehensive clinical 1.5T MRI-Linac dataset. The neural network exhibited correct modelling of magnetic field effects in water phantom and patient settings. Using a gamma criterion of 3 mm/3%, median γ-PR of 97.7% and 97.1% could be achieved for all treatment plans and individual segments of the test dataset respectively. The calculation time was reduced from several hours (full MC simulation) to seconds (ours). Therefore, this method offers a valid option for fast secondary dose verification.

In this work, the significance of using comprehensive test data became clear. While trained on a heterogeneous dataset, the performance of the network differed on the five test data subsets. Highest γ-PRs were obtained for the prostate test dataset, comparable to the results presented by Kontaxis et al. [19] and Tsekas et al. [24], of which the network layout was adopted. Kontaxis et al. only incorporated prostate tumor patients, with fixed beam setups and without magnetic field, which should reduce the complexity of the task. Nevertheless, segments with low γ-PR (3 mm/3%, minimum < 65%) were observed in this study. This effect was also visible in our work with a minimum γ-PR of 68.4% (3 mm/3%) in the prostate test dataset. Very few segments with low acceptance rates were observed in the four other test datasets as well (Fig. 2), not showing striking similarities in their properties. Single segments with lower agreement (minimum 2 mm/2% γ-PR: 87.7%) also occurred in the work of Pastor-Serrano et al. [20], despite them showing a median 2 mm/2% γ-PR of 98.1% and 96.9% for irradiation segments of two Pelvic and Lung test datasets. This could indicate a high potential of transformer-based architectures, but the small size of the test set of 584 irradiation segments and the lower complexity due to the absence of a strong external magnetic field must be considered.

A recent study [24] considered the magnetic field and radiotherapy plans for rectal cancer and oligometastatic lymph nodes located in the abdominal region. Again, fixed beam angles were used. Their predictions showed a mean acceptance rate for treatment plans of 99.4% and 96.1% for the 3 mm/3% and 2 mm/2% gamma criterion respectively and therefore seem comparable with the agreement of the corresponding entities prostate and liver in this work (98.7%, 92.5%), considering the higher complexity of our datasets. However, Tsekas et al. [24] reported a γ-PR of 82.2% when using the 1 mm/1% criterion, which contrasts with the mean agreement of 68.1% obtained in this study. Reasons for that might be the sparser initial dose grid used for calculation of the dose and the less heterogeneous dataset [24].

A key aspect of our work was to test the suitability of the approach for tumor regions close to tissue surfaces and complex anatomies. Therefore, the partial breast irradiation dataset and the HNC dataset were added, and the Sobel kernel-based loss function was developed. Both datasets seem to be unique in the context of deep neural network driven dose modelling at an MRI-Linac. The network performance decreased on those datasets, compared to the prostate and liver data. One reason might be the challenges of (air-)tissue interfaces for dose calculation in magnetic fields. Nevertheless, the median γ-PRs were above 96% (3 mm/3%) for treatment plans and 96.1% and 95.3% for individual segments. Using stricter criteria the pass rates decreased more drastically than in the liver and prostate test datasets.

The out of domain lymph node test dataset showed the highest standard deviation of all test sets. When tested with a 3 mm/3% criterion, the performance of the network on this dataset is not significantly different from the other test datasets for whole treatment plans but is significant for individual segments. This indicates the generalizability of this network on a treatment plan scale.

In the water phantom setting key features of dose distributions in presence of a strong magnetic field got modelled correctly. Despite being trained on patient data, the modelled dose distribution aligned well with the MC simulation and the measurements. Only in the in-line direction deviations smaller than one slice thickness of the original dataset become apparent. This could be due to the definition of isocenters between two slices in the training data.

To further increase the robustness of the network, it could be beneficial to include fast dose calculation as an input to the neural network, as done in denoising approaches [4], [17], [18]. On the downside, additional preprocessing steps are needed, potentially limiting the real time capabilities of the approach. As indicated by Pastor-Serrano et al. [20], transformer-based model architectures may help to incorporate long distance relationships. A valid uncertainty estimation could help to identify situations with low model performance, could improve the training via an active learning approach or add dose ranges to model predictions. Another future step could be to model dose distributions directly on MRI data, skipping pseudo-CTs used in online adaptive, MRI-guided workflows [32].

In conclusion, we trained and evaluated a 3D U-Net based neural network successfully on a highly clinical and comprehensive 1.5T MRI-Linac dataset. Magnetic field effects were modelled well and systematic errors in the modelled dose couldn’t be observed. The model performance was dependent on the tumor entity of the test data, with best agreement achieved in prostate and liver cancer cases. Dose calculation at tissue-interfaces, as included in the partial breast irradiation dataset, and complex anatomies, as included in the HNC dataset, lead to decreasing agreement, while still maintaining high median acceptance rates, especially for a 3 mm/3% gamma criterion. As few individual segments showed unsatisfactory agreement with the MC simulated ground truth data, we emphasize the need for comprehensive, heterogenous clinical datasets and the need for implementation of uncertainty estimation methods.

## CRediT authorship contribution statement

**Moritz Schneider:** Methodology, Software, Formal analysis, Investigation, Writing – original draft, Visualization. **Simon Gutwein:** Methodology, Software, Investigation, Writing – review & editing. **David Mönnich:** Resources, Writing – review & editing. **Cihan Gani:** Resources, Writing – review & editing. **Paul Fischer:** Methodology, Writing – review & editing. **Christian F. Baumgartner:** Conceptualization, Methodology, Resources, Writing – review & editing, Supervision. **Daniela Thorwarth:** Conceptualization, Resources, Writing – review & editing, Supervision.

## Declaration of competing interest

The authors declare the following financial interests/personal relationships which may be considered as potential competing interests: The Department of Radiation Oncology Tübingen receives financial and technical support by Elekta, Philips, Dr. Sennewald Medizintechnik, TheraPanacea, Brainlab and PTW Freiburg in the context of research cooperations. Cihan Gani received travel and speaker honoraria from Elekta.
