# Supplementary material for: Development and comprehensive clinical validation of a deep neural network for radiation dose modelling to enhance magnetic resonance imaging guided radiotherapy
Source: Phys Imaging Radiat Oncol. 2025 Feb 7;33:100723. doi: 10.1016/j.phro.2025.100723 (PMC11908596; doi:10.1016/j.phro.2025.100723)
Supplement: Supplementary Data 1 [file mmc1.docx]

**Supplementary materials**

*A Definition of the customized loss function*

The customized loss function, used for the subsequent part of the training is depicted in equations 1-5. It is designed to stress areas with high gradients in the dose domain. Therefore, an additional term was added to the standard squared error loss. It scales the squared error with a three-dimensional convolutional kernel in the dose domain. The three-dimensional kernel matrices used to identify the gradient in the x,y and z-direction respectively, are depicted in equation 3,4 and 5. They follow the idea of gradient detection using a Sobel kernel [1].

| $L=\sqrt{\frac{1}{n}\sum_{i=0}^{n} \left( \left( y_{i}-\hat{y_{i}} \right)^{2}+0.1 (\left( y_{i}-\hat{y_{i}} \right)^{2}\times G\left( y_{i} \right)) \right)}$ | (1) |
| --- | --- |
| $G\left( y \right)=\sqrt{{G_{x}}^{2}+{G_{y}}^{2}+{G_{z}}^{2}}$ | (2) |
| $G_{x}=\left( \left( \begin{matrix} 1 & 2 & 1 \\ 0 & 0 & 0 \\ -1 & -2 & -1 \end{matrix} \right),\left( \begin{matrix} 2 & 4 & 2 \\ 0 & 0 & 0 \\ -2 & -4 & -2 \end{matrix} \right),\left( \begin{matrix} 1 & 2 & 1 \\ 0 & 0 & 0 \\ -1 & -2 & -1 \end{matrix} \right) \right)*y$ | (3) |
| $G_{y}=\left( \left( \begin{matrix} 1 & 2 & 1 \\ 2 & 4 & 2 \\ 1 & 2 & 1 \end{matrix} \right),\left( \begin{matrix} 0 & 0 & 0 \\ 0 & 0 & 0 \\ 0 & 0 & 0 \end{matrix} \right),\left( \begin{matrix} -1 & -2 & -1 \\ -2 & -4 & -2 \\ -1 & -2 & -1 \end{matrix} \right) \right)*y$ | (4) |
| $G_{z}=\left( \left( \begin{matrix} 1 & 0 & -1 \\ 2 & 0 & -2 \\ 1 & 0 & -1 \end{matrix} \right),\left( \begin{matrix} 2 & 0 & -2 \\ 4 & 0 & -4 \\ 2 & 0 & -2 \end{matrix} \right),\left( \begin{matrix} 1 & 0 & -1 \\ 2 & 0 & -2 \\ 1 & 0 & -1 \end{matrix} \right) \right)*y$ | (5) |

**References:**

[1] Sobel I, Feldman G. A 3x3 isotropic gradient operator for image processing. A Talk at the Stanford Artificial Project in 1968 1968;1968:271–2.

*B Performance without the subsequent training*

The model trained in the first training step, using the RMSE loss function only, showed some increased errors around dose gradients. To improve the performance in such areas, a subsequent training, starting from that model, was performed emphasizing the dose gradients using a gradient based scaling of the loss (supplementary materials A).

Table S1 shows the evaluation of the model trained solely with the RMSE loss on the irradiation segments test dataset:

Table S1: 𝛾-PRs of individual segments from the test datasets, evaluated with different criteria; predictions with RMSE trained model only.

| *Dataset* | ***Prostate*** | | | ***Liver*** | | | ***Breast*** | | | ***HNC*** | | | ***Lymph node*** | | |
| --- | --- | --- | --- | --- | --- | --- | --- | --- | --- | --- | --- | --- | --- | --- | --- |
| *Criterion* | *3 mm/3%* | 2 mm/2% | *1 mm/1%* | *3 mm/3%* | 2 mm/2% | *1 mm/1%* | *3 mm/3%* | 2 mm/2% | *1 mm/1%* | *3 mm/3%* | 2 mm/2% | *1 mm/1%* | *3 mm/3%* | 2 mm/2% | *1 mm/1%* |
| *Median [%]* | 99.0 | 93.3 | 66.9 | 97.2 | 87.9 | 58.4 | 93.5 | 82.3 | 55.0 | 94.2 | 83.7 | 56.7 | 93.9 | 80.4 | 49.2 |
| *Mean [%]* | 97.5 | 90.3 | 65.4 | 95.3 | 85.9 | 59.2 | 92.2 | 80.3 | 53.1 | 92.4 | 82.0 | 55.5 | 90.8 | 78.3 | 50.7 |
| *Min [%]* | 81.6 | 59.8 | 26.1 | 59.6 | 42.4 | 18.3 | 65.9 | 45.3 | 18.0 | 51.8 | 38.3 | 18.7 | 50.9 | 34.8 | 17.1 |
| *Max [%]* | 100.0 | 99.5 | 90.1 | 100.0 | 99.3 | 95.0 | 99.8 | 96.4 | 81.2 | 99.4 | 96.5 | 83.0 | 100 | 99.8 | 87.1 |
| *STD [%]* | 3.4 | 8.0 | 13.1 | 5.4 | 10.0 | 13.3 | 6.0 | 10.5 | 12.4 | 5.9 | 9.5 | 11.7 | 9.0 | 13.1 | 13.8 |

The subsequent training using the customized loss function led to an increased mean and median agreement in all datasets. Most prominent is the improvement in the breast dataset with an increase in mean agreement of 2.2%, 4.2% and 4.5% for the three criteria respectively. The least increase could be observed in the prostate test dataset. There the subsequent training led to an increase in mean agreement of 0.4%, 1.6% and 3.8% for the three criteria respectively.
